# Cutaneous Leishmaniasis in a Recreational Cave Diver After Travel to México

**DOI:** 10.7759/cureus.16896

**Published:** 2021-08-04

**Authors:** Alfredo Siller Jr., Benjamin A Garcia, Evan J Kipp, Michael Lee, Stephen Tyring

**Affiliations:** 1 Dermatology, Center for Clinical Studies, Webster, USA; 2 Dermatology, University of Texas Medical Branch, Galveston, USA; 3 Veterinary Medicine, College of Veterinary Medicine, University of Minnesota, Saint Paul, USA; 4 Dermatology, MedDerm Associates Inc, San Diego, USA; 5 Dermatology, Center for Clinical Studies, Houston, USA

**Keywords:** cutaneous leishmaniasis, infectious and parasitic diseases, leishmania mexicana, phlebotominae, tropical dermatology

## Abstract

Cutaneous Leishmaniasis due to *Leishmania mexicana* is a common cause of New World protozoal infections endemic to southern Mexico and now the United States (US). We present a case of a 72-year-old male who became infected with cutaneous *L. mexicana *while participating in numerous diving excursions in the flooded limestone caves, commonly referred to as *cenotes, *in the Yucatán Peninsula. This unique case of adventure tourism highlights cave diving in endemic regions of leishmaniasis as a possible new risk factor for the acquisition of this disease. We also discuss increasing autochthonous cases of cutaneous leishmaniasis and the different barriers to treatment that occur with this disease.

## Introduction

Transmitted by phlebotomine sand fly vectors, leishmaniases are a group of globally distributed protozoal diseases capable of presenting with a broad range of clinical syndromes [1]. Moreover, the most common form of the disease - cutaneous leishmaniasis (CL) - is itself characterized as having numerous dermal manifestations [2]. Leishmaniasis remains a highly relevant and neglected parasitic disease throughout the neotropics. Although conventionally viewed in the United States (US) as a travel-associated disease, it has become increasingly apparent that autochthonous transmission of CL caused by *Leishmania mexicana* does occur across portions of the southern US [3,4]. Additionally, treatment for CL is often difficult as many treatment options are limited in cost, toxicity, and FDA approval.

## Case presentation

**Figure 1:**
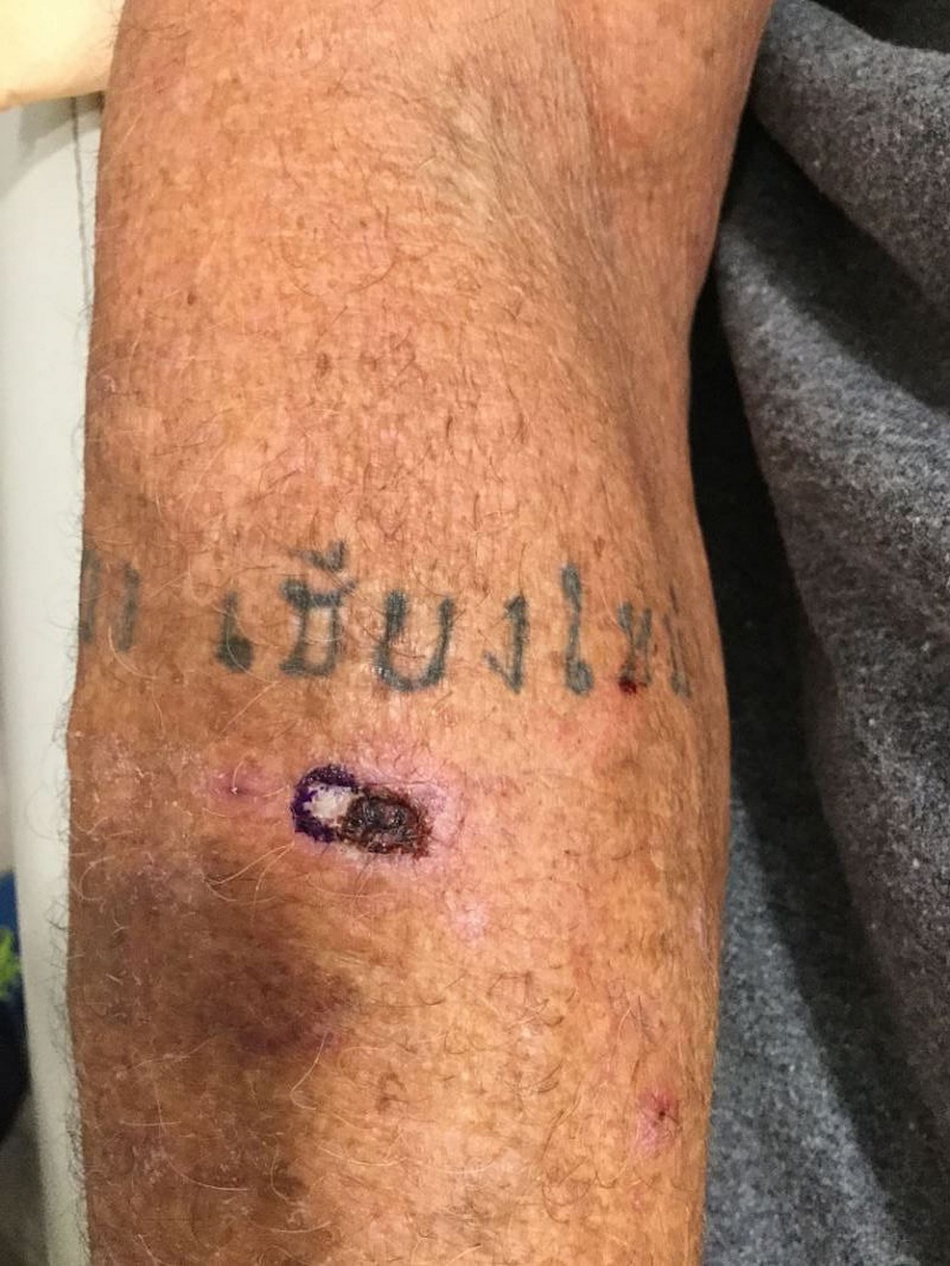
Dermal crusted ulceration on the patient's right forearm diagnosed as cutaneous leishmaniasis taken approximately six months after initial lesion onset.

**Figure 2:**
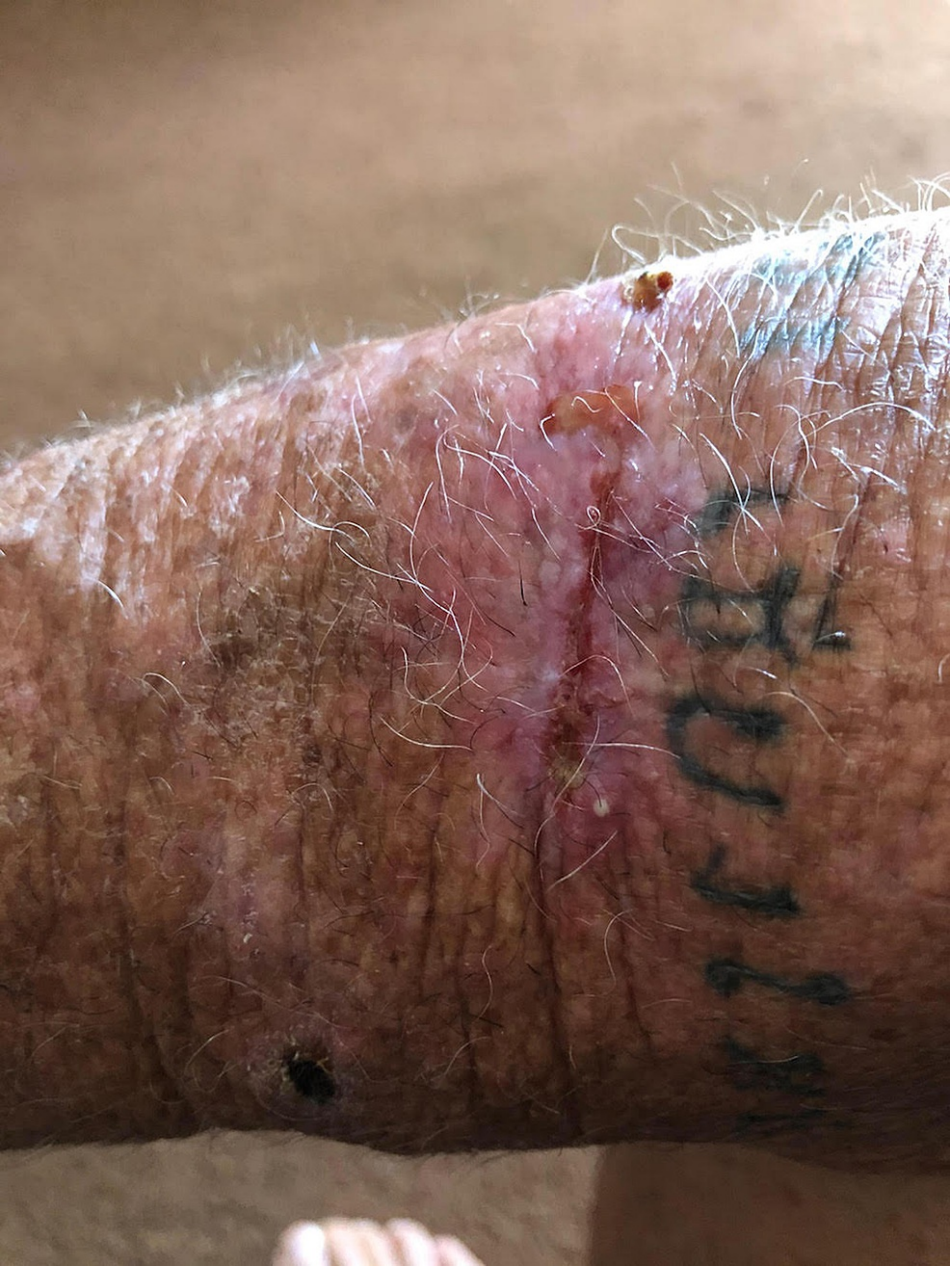
Dermal crusted lesion on the patient's right forearm diagnosed as cutaneous leishmaniasis taken approximately 10 months after lesion onset.

A 72-year-old male was referred to a dermatology clinic in San Diego, CA, for treatment of a solitary crusted ulceration on his right forearm present for three months. The lesion was approximately 1 cm in diameter and described as a “bug bite” that erupted following a trip to the Yucatán Peninsula in the state of Quintana Roo, México, two months prior. An outside physician performed a shave biopsy, which revealed the presence of protozoa consistent with *Leishmania* amastigotes upon histologic examination. Treatment with the antiprotozoal chemotherapy miltefosine was discussed; however, the patient ultimately deferred this treatment option due to the high out-of-pocket cost. As an alternative, the patient elected to initiate treatment with oral fluconazole (200 mg daily for six weeks); unfortunately, after completion of this course, the lesion remained persistent and non-healing. Examination of the lesion at this time showed 1-cm ulceration with erythematous raised borders and smaller adjacent erythematous, eroded round papules (Figures 1, 2).

**Figure 3:**
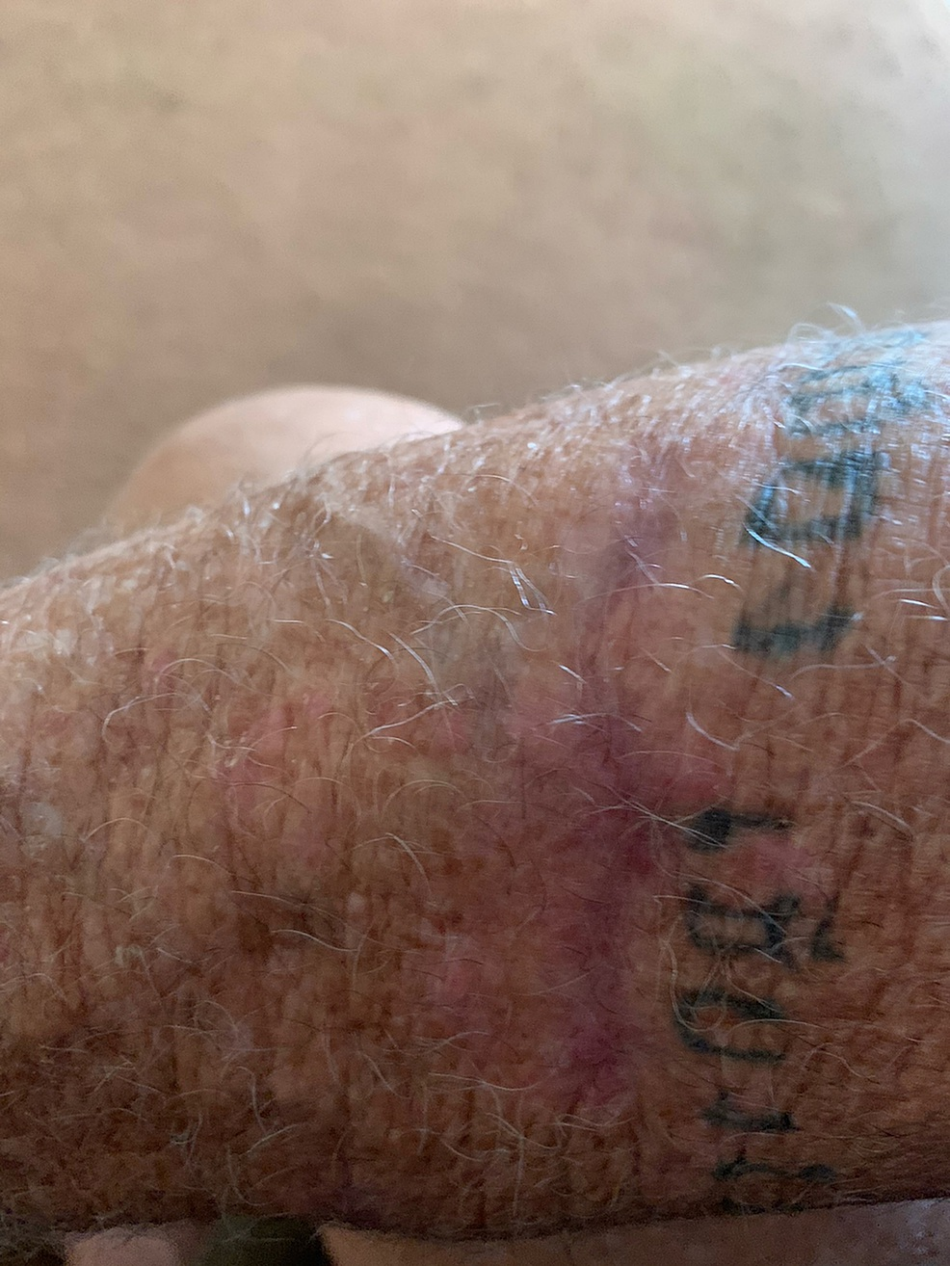
Dermal crusted ulceration on the patient's right forearm diagnosed as cutaneous leishmaniasis taken approximately 14 months after lesion onset.

**Figure 4:**
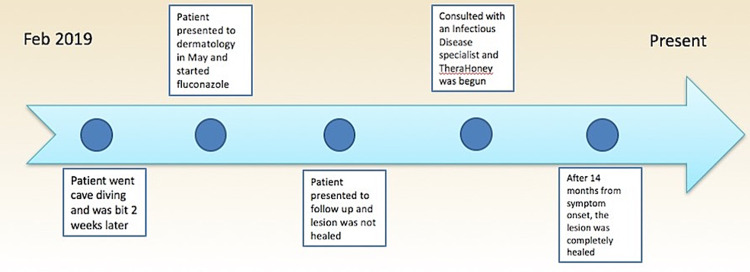
Timeline of events from lesion onset to resolution.

A punch biopsy of the edge of the crusted ulceration was sent to the U.S. Centers for Disease Control and Prevention (CDC) for diagnostic confirmation. *Leishmania *amastigotes were observed in clinical specimens through culture and microscopy, and PCR/DNA sequencing determined the infecting species as *L. mexicana.* The patient was referred to an infectious disease specialist who recommended only symptomatic care with TheraHoney®, as cutaneous lesions caused by *L. mexicana* are frequently self-limiting and known to resolve spontaneously, generally within three to nine months [2]. At follow-up, 14 months from the onset of his lesion, the patient reported his ulceration had completely healed after symptomatic care with TheraHoney®, albeit with a distinct scar (Figure 3). A timeline of the patient's infection is also shown below (Figure 4).

## Discussion

**Figure 5:**
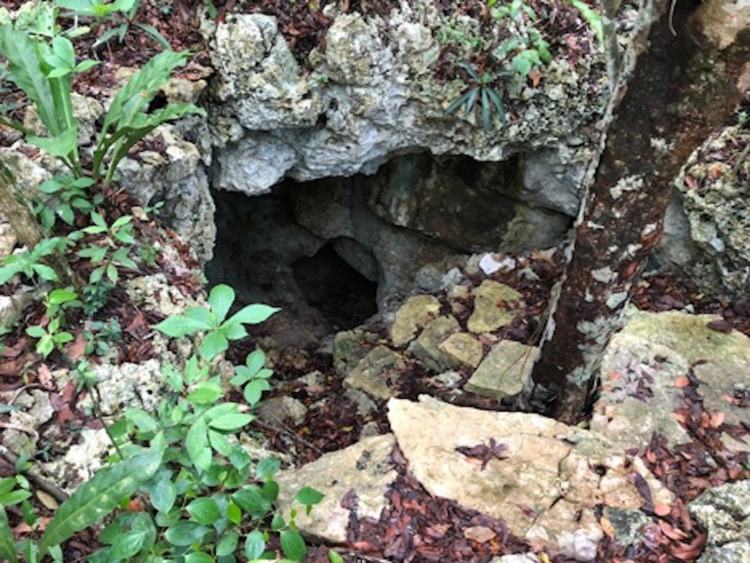
Cenote entrance in Quintana Roo, México, where the patient reported cave diving and believes his exposure to L. mexicana likely occurred. Note the surrounding decaying debris of leaf litter and numerous moist rock crevices that form an advantageous microhabitat for the phlebotomine sand fly vectors of leishmaniasis.

Here, we describe a case of New World CL in a patient from California who acquired infection with *L. mexicana* on the right forearm while cave diving in Mexico. During his time in México, the patient described participating in numerous diving excursions to the flooded limestone caves commonly referred to as cenotes. Moreover, he recalled receiving repeated bites from unidentified insects while outside the cave entrances at multiple dive sites (Figure 5).

These karstic cave systems contain humid microhabitats that consist of decaying debris, creating an environment that maintains a diversity of sand fly vector species by allowing the eggs and larvae of the flies to thrive [5]. This case is of particular interest for several reasons. To our knowledge, no published cases of* L. mexicana*, in California, have been reported in the literature, nor have any cases reported leishmaniasis infection acquired from cave exposure. This case highlights a unique opportunity to discuss cave exploration, in endemic regions, as a high-risk exposure activity for the acquisition of infection with leishmania.

CL due to* L. mexicana* is a common cause of New World protozoal infections endemic to southern Mexico and now the US [2]. Kipp et al. noted that most cases of human leishmaniasis previously identified in the US have occurred among persons who have lived and traveled abroad [6]. However, a growing number of autochthonous CL cases have been reported in the US in recent years, especially in CLthe south-central U.S. where it is acquired endemically more frequently than it is via travel [3,4,7]. In contrast to Old World species infections, cases of leishmaniasis caused by* L. mexicana*, may have a diverse clinical presentation including papular, nodular, or ulcerative dermatosis. Reasons for this include heterogeneity of Leishmania species that cause disease; varying clinical manifestations and response to treatment depending on geographic acquisition of infection; lack of high-quality clinical trials for optimal medical management; and, in the United States, affordability, toxicity, and availability of medications [7].

Despite the increasing incidence of leishmaniasis in the US, standardized treatment options continue to be a challenge. Treatment options for CL in the US are limited in cost, toxicity, and FDA approval. Currently, Miltefisone is FDA approved for CL but is extremely expensive. Additionally, due to its teratogenic and gastrointestinal side effects, its use has become limited [8]. Amphotericin B and liposomal amphotericin B have both been shown to have some efficacy in regard to CL, but they both are expensive and require hospitalization [8]. Pentavalent antimonials are usually first-line treatments for CL; however, they are only available for use in the US through the CDC and are not FDA approved for CL. These drugs also have a high side effect profile and due to their parenteral route and duration of treatment can cause adherence problems [9]. Most clinical trials for CL have been designed and reported poorly, resulting in a lack of evidence for potential beneficial treatments [7]. This may be attributed to a possible lack of financial incentive for pharmaceutical companies to invest in the development of drugs for a disease that affects people that do not have financial resources, in addition, proper follow up is problematic as this disease occurs mainly in remote areas where many studies have been affected by a considerable number of loss to follow-ups [10]. Because of the increased incidence of autochthonous CL cases in the US, and with leishmaniasis as a category 1 emerging and uncontrolled disease [10], there is a growing interest for large well-conducted studies and standardization of future trials to improve vector control, diagnostics, and therapeutics to contain further incidence and morbidity [10].

## Conclusions

Both healthcare providers and travelers should be aware of the potential risk that cave diving and other increasingly popular forms of “adventure tourism” may represent in promoting human-sandfly contact. Additionally, we urge providers with suspect cases of CL to seek diagnostic assistance through the CDC to facilitate identification of infecting *Leishmania* spp., to direct appropriate antiprotozoal treatment, and to epidemiologically differentiate between autochthonous and travel-associated cases. 
